# Resilience to Stigma in Medical, Social, and Employment Contexts Among People Who Inject Drugs in Rural Ohio: Adapting the 10-Item Connor-Davidson Resilience Scale

**DOI:** 10.1007/s10461-025-04915-4

**Published:** 2025-10-28

**Authors:** Madison N. Enderle, Rebecca H. Neiberg, Stacy M. Endres-Dighe, Nisha Gottfredson O’Shea, Vivian F. Go, William C. Miller, Kathryn E. Lancaster

**Affiliations:** 1 Division of Public Health Sciences, Department of Implementation Science, Wake Forest University School of Medicine, Winston-Salem, NC USA; 2 Division of Public Health Sciences, Department of Biostatistics and Data Science, Wake Forest University School of Medicine, Winston-Salem, NC USA; 3 Division of Epidemiology, College of Public Health, The Ohio State University, Columbus, OH USA; 4 Applied Public Health Research Center, RTI International, Research Triangle Park (RTP), NC USA; 5 Division of Behavioral Health and Wellbeing, RTI International, Research Triangle Park (RTP), NC USA; 6 Department of Health Behavior, Gillings School of Global Public Health, The University of North Carolina at Chapel Hill, Chapel Hill, NC USA; 7 Department of Epidemiology, Gillings School of Global Public Health, The University of North Carolina at Chapel Hill, Chapel Hill, NC USA

**Keywords:** Substance use, HIV prevention, Stigma, Resilience, Rural

## Abstract

People who inject drugs (PWID) experience stigma related to drug use and HIV prevention, which can impede engagement with the HIV prevention continuum. Resilience may buffer against stigma’s harmful effects, but limited research has examined how resilience operates across different social contexts and stigma types among PWID. Using qualitative interviews and cognitive interviews, we adapted the 10-item Connor-Davidson Resilience Scale to measure resilience to drug use-related stigma and HIV prevention-related stigma among PWID in rural Appalachian Ohio in three contexts: medical, social, and employment. To validate the scale adaptations, we administered a quantitative survey. We assessed internal consistency using Cronbach’s alpha and fit Poisson regression models to test the association between high resilience and pre-exposure prophylaxis (PrEP) awareness and overdose response training. Among the 250 PWID who completed the quantitative survey, the six resilience scales demonstrated strong internal consistency. Examining frequency distributions, Bland–Altman plots, and factor analysis, we found the resilience responses varied by stigma type and context. Results of regression models indicated high resilience to HIV prevention-related stigma in the medical context was associated with PrEP awareness (adjusted prevalence ratio [aPR]: 1.37; 95% CI 1.05, 1.80) and overdose response training (aPR: 1.31; 95% CI 1.07, 1.61). Our study provides a novel approach to measuring context-specific resilience to stigma among PWID. Findings highlight the importance of tailoring interventions to both individual resilience and structural stigma, particularly in medical settings. Enhancing resilience and reducing stigma may support greater engagement with HIV prevention and harm reduction services in rural communities.

**Supplementary Information:**

The online version contains supplementary material available at 10.1007/s10461-025-04915-4.

## Introduction

Drug use-related stigma and HIV prevention-related stigma threaten engagement with the HIV prevention continuum among those most in need—people who inject drugs (PWID) [1–7]. Among PWID, sharing of injection needles and equipment for drug use has led to a dramatic rise in hepatitis C, the harbinger for HIV, particularly in rural Appalachian settings [8–10]. Appalachia was hard hit by the opioid epidemic, likely due to a combination of socioeconomic challenges and pill mills, and the region continues to struggle with the associated impacts, including injection drug use and need for hepatitis C and HIV treatment and prevention [11–13]. Engaging PWID in timely HIV prevention may be critical to averting an HIV outbreak yet is often complex due to pervasive stigma. Understanding and addressing the stigma faced by PWID is essential for overcoming barriers to their participation in HIV prevention efforts.

In Appalachia, drug use-related stigma has led to PWID avoiding healthcare due to feeling internalized shame of their drug use and anticipated fear of disrespectful treatment from healthcare providers [5, 7, 11, 14, 15]. HIV prevention-related stigma is less recognized but equally pervasive and likely overlapping with drug use-related stigma in rural settings [6, 16–18]. HIV prevention-related stigma among PWID hinders HIV testing due to concerns about disclosure, particularly in small, close-knit social networks [2, 19, 20]. Pre-exposure prophylaxis (PrEP) is also often perceived negatively among PWID as it may lead to others’ assumptions of HIV status or HIV risk behaviors [21–23]. Until we gain further understanding of drug use-related and HIV prevention-related stigma and improve engagement in the HIV prevention continuum, PWID will remain at significant risk for HIV.

PWID can counteract stigma through resilience. Resilience is a process in which individuals positively adapt to withstand adversity and avoid harmful events that result in suboptimal health outcomes [24–28]. Resiliency can be enhanced through developing self-efficacy, coping skills, and social support [24, 27]. Resilience interventions which focus on cultivating self-efficacy and social support are effective for overcoming stigma and improving care among marginalized populations, like people living with HIV [29, 30]. PWID would likely benefit from strengthened self-efficacy and social support to improve healthcare utilization [27, 31–33]. However, the mechanisms of the resilience process and potential effectiveness are mostly unknown among PWID. An enhanced understanding of resilience mechanisms among PWID is needed to advance interventions promoting resilience. Further, understanding PWID’s resilience, including identifying specific settings where PWID’s resilience is insufficient to overcome systemic stigma, could inform interventions to reduce systemic stigma and barriers to care, reducing the level of resilience needed to navigate the healthcare system [34].

To our knowledge, no quantitative measures of resilience to stigma are tailored explicitly for PWID. The existing 10-item Connor-Davidson Resilience Scale (CD-RISC-10), which has excellent reliability and validity, captures general coping strategies and personal qualities which broadly represent resilience among the non-marginalized population [25, 35]. But the scale does not address resilience to particular forms of stigma. Resilience is a dynamic process that changes over time and varies by population and sources of stigma [27, 32, 33]. A resilience scale ideally should not just capture one’s ability for general resiliency but one’s ability to withstand stigma as part of their unique environment [32]. Without a tailored measure for PWID, the ability to assess resilience to drug use-related and HIV prevention-related stigma and differentiate it from general skills or experiences is complicated [31]. Measuring resilience among PWID can inform accessible interventions to support resilience, reduce systemic stigma and barriers, and, ultimately, improve engagement with the HIV prevention continuum.

To inform future intervention outcomes for enhancing the HIV prevention continuum, we conducted in-depth interviews, cognitive interviews, and quantitative surveys to adapt the CD-RISC-10 and develop and validate a PWID Resilience Scale to measure resilience to drug use-related and HIV prevention-related stigmas encountered in a variety of contexts among PWID in rural Appalachia.

## Methods

### Study Design and Scale Development Overview

This study, referred to here forward as the *Measuring Resilience* study, was a community-based, cross-sectional research program conducted in rural Appalachian Ohio. Of the thirty-two Ohio Appalachian counties, we chose to recruit from six counties which were among the top 5% of counties in the United States most vulnerable to an HIV outbreak [36]. These counties are predominately non-Hispanic white and are classified as economically “distressed” or “at risk” by the Appalachian Regional Commission [37–39].

We used a mixed methods research approach to inform the development and validation of a PWID Resilience Scale to measure resilience to drug use-related and HIV prevention-related stigma among PWID in rural Ohio. Specifically, the scale was developed in three phases including: (1) semi-structured, in-depth qualitative interviews to explore how mechanisms of resilience are displayed, shared, and enacted to counter drug use-related and HIV prevention-related stigmas; (2) cognitive interviews to explore levels of resilience to drug use-related and HIV prevention-related stigmas; and (3) a quantitative survey to validate the PWID Resilience Scale developed via the in-depth and cognitive interview processes described above.

### Phase 1: Semi-structured In-Depth Qualitative Interviews

We conducted semi-structured, in-depth qualitative interviews to investigate the interplay of drug use-related and HIV prevention-related stigmas, and how resilience processes are displayed, shared, and enacted to counter stigma. We recruited participants within syringe services programs (SSPs), health clinics, and resource centers in the six study counties and used purposive sampling to ensure variation in gender and HIV testing history. To be eligible, participants were required to meet the following criteria: (1) 18 years of age or older, (2) residing in one of these six study counties, (3) never received an HIV test or never tested positive if they had been tested, and 4) used injection drugs within the two weeks prior to recruitment.

One-on-one qualitative interviews were conducted between October 2021 and July 2022. Study staff provided study information, obtained verbal informed consent, and administered the qualitative interviews. Interviewers were extensively trained in non-judgmental techniques, various approaches for building rapport, and probing techniques to avoid relying on direct questioning. Interviews were completed in English, lasted between 1 and 2 h and, due to the ongoing COVID-19 pandemic, were conducted remotely, via Zoom. We continued enrolling participants until we met theoretical saturation. Of the 39 PWID who we invited to participate, 1 was not eligible and 3 did not provide informed consent. The remaining 35 individuals were enrolled and completed the interview. A $25 electronic gift card was provided to participants as compensation for their time.

All interviews were audio-recorded and transcribed verbatim. Qualitative methodology used during phase 1 of the *Measuring Resilience* study is described elsewhere [40]. In brief, the study team used thematic analysis to identify, analyze, and interpret patterns within the interview transcripts. The codebook was developed using both deductive and inductive approaches. Specifically, the initial codebook was based on Harper et al.’s core resilience processes [24]. Two study team coders reviewed a subset of five transcripts, line-by-line, allowing for codes to emerge inductively from the data [41]. The initial code definitions were then refined, and new codes created, until emerging themes were exhausted, resulting in a finalized codebook. The final codes were then applied to all transcripts and inter-coder reliability was evaluated via double coding for 14% of all transcripts, with a Cohen’s kappa of 0.81. N*VIVO 12.0 qualitative data analysis software was used to manage data and code transcripts.

Results from the in-depth qualitative interviews were used to inform the adaptation of the resilience scale items (Supplementary Tables S1 and S2). Specifically, the study team (including a Portsmouth city health department partner) reviewed the results and identified common stigmatizing scenarios (i.e., injection drug use and HIV prevention) which were experienced by Appalachian PWID in three unique contexts (i.e., social, employment, and medical). We then identified quotes which exemplified the stigmatizing experiences in each context and employed these quotes in the development of six scenarios (Supplementary Table S1). We intentionally selected gender-neutral names for use in each scenario to ensure applicability for both male and female participants.

Next, we developed a cognitive interview instrument to measure resilience to drug use-related and HIV prevention-related stigmas among PWID. This instrument employed the PWID Resilience Scale, which consisted of the six in-depth interview-informed scenarios and an adapted version of the CD-RISC-10 [35]. Each adapted item corresponded to a CD-RISC-10 item, ensuring the three resilience dimensions covered by the CD-RISC-10 (grit, unflappability, acceptance) out of the original five CD-RISC domains would also be included in the PWID Resilience Scale (Supplementary Table S2) [25, 35].

### Phase 2: Cognitive Interviews

We conducted semi-structured cognitive interviews to (1) explore levels of resilience to drug use-related and HIV prevention-related stigmas shared among PWID in rural Appalachian Ohio and (2) confirm the clarity and conceptual relevancy of the PWID Resilience Scale for the rural Ohio context. Participant recruitment strategies and eligibility criteria for phase 2 cognitive interviews mirrored that of phase 1. Study staff provided study information, obtained written informed consent, and administered the cognitive interview. A total of 15 one-on-one cognitive interviews were completed with PWID between September and December 2022. A $25 gift card was provided to participants as compensation for their time.

Interviews were conducted in English, audio-recorded, and lasted approximately 30 min. Interview questions consisted of the six scenarios and the 10-item PWID Resilience Scale. Additionally, trained study staff probed after each scenario to ensure relevance of the scenario and the items and to assess ease of answering the items (e.g., Can you tell me about a time when something like this happened to you?; What question was most difficult to answer? Why?). Immediately following each cognitive interview, the interviewer wrote detailed summary notes. Summary notes focused on how participants determined their answers, recalled information, comprehended items, and explained items in their own words. The rest of the study team was subsequently debriefed to review findings for each survey question and discuss decisions for item tailoring to enhance competency.

Debriefs, summary notes, and review of audio-recordings of the cognitive interviews demonstrated the participants had a good understanding of each item and that the scenarios were conceptually relevant to participants. We retained all six scenarios and 10 items in the final version of the PWID Resilience Scale. The *Measuring Resilience* study team used preliminary findings from the in-depth and cognitive interviews (qualitative phase 1 and 2, respectively) to inform the development of a quantitative survey to validate and apply the PWID Resilience Scale.

### Phase 3: Quantitative Survey

We recruited participants using peer-referrals through non-identifying codes. Peer referrals are an effective recruitment method among vulnerable, hard-to-reach populations, like PWID in rural communities [42]. Initial participants, “seeds,” were purposively chosen based on field staff’s prior experience with the community. Each eligible participant who enrolled could refer up to four peers. To be eligible, participants must have met the following criteria: (1) be 18 years of age or older, (2) live in one of these six study counties, (3) have never received an HIV test or never tested positive if they had been tested, and (4) used injection drugs within 30 days of recruitment. Enrollment, written informed consent, and data collection for the quantitative survey were conducted in-person.

A total of 250 surveys were administered from March to October 2023, using computer-assisted (REDCap) self-interview. Participants completed the survey in a private location within a study facility located in a single study county near community service sites such as SSPs and health clinics. Survey administration took approximately 1–2 h and consisted of questions on sociodemographic, substance use, injection behavior, sexual history, HIV knowledge, stigma, and the PWID Resilience Scale. Participants were compensated $50 for participation in the survey and an additional $25 (not to exceed a total of $100) for each recruit who appeared at the study site and was eligible to participate.

### Statistical Analyses

#### Demographic Characteristics

We summarized demographic characteristics as median (interquartile range) for continuous and frequency (percent) for categorical measures.

#### Comparison of Scales Across and Within Stigmas and Contexts

To investigate implications and potential loss of information from combining responses across and within stigma type (drug use-related or HIV prevention-related) and context (social, employment, or medical), we considered both item-level and summed item scale-level comparisons. We compared item level responses with stacked bar charts and Spearman non-parametric rank correlations between contexts within stigmas. We examined Bland–Altman plots of difference and bias within stigmas and within contexts. We considered Cronbach’s alpha tests for consistency for each of the six scenarios.

To identify factors within each stigma-context combination, we conducted separate exploratory factor analysis (EFA) with oblique Promax rotation to allow for non-normal distribution of items. We split the data into three random samples to assess replicability of EFA between the first and second subset and determine robustness of findings [35, 43]. With the third random subset, we planned to conduct confirmatory factor analysis (CFA) though we were ultimately unable to complete this given the results of the EFA [35]. We also compared the EFA results of the subsets to the EFA results of the full sample.

#### Regression Analysis

We compared unadjusted frequencies and percents of awareness of PrEP (yes or no/don’t know) and taken overdose training (yes or no/don’t know) with chi-square tests and then further analyzed using a Poisson regression with robust error variance to estimate prevalence ratios (PR) with 95% confidence intervals (CI) of resilience [44, 45]. We examined each of the six stigma-context scenarios of resilience as key independent variables in the models as high versus low resilience categories (sum of items ≥25, <25) according to prior work [46, 47]. All models adjust for a parsimonious set of covariates based on the literature: respondents’ sex, age category (≥30 years, <30 years), high school completion, living with partner, and unstable housing in last 6 months [48–50].

All analyses were conducted in SAS9.4 (SAS Institute, Inc, Cary, NC). Boxplots were constructed using the ‘ggplot2’ package in RStudio 2023.03.0+385 [51].

## Results

### Study Population Characteristics

All participants (n = 250) resided in a single study county. Participants ranged in age from 20 to 68 years (median = 38), half identified as female (n = 125), and nearly all were white (90%, n = 223) and non-Hispanic (99%, n = 247 [Table 1]), consistent with the county population. Nearly a third did not complete high school (32%, n = 78), about one-quarter had a full- or part-time job (27%, n = 67), and 57% had experienced unstable housing in the past 6 months (n = 142). Most participants reported an opioid as their current drug of choice (68%, n = 166).

**Table 1:** Characteristics of n = 250 PWID from rural Appalachian Ohio who completed quantitative surveys March–October 2023

	n	(%)
Age, median (IQR)	38	(32,46)
Sex/gender		
Female	125	(50.0)
Male	125	(50.0)
Race		
White	223	(90.3)
African American or Black	10	(4.05)
American Indian or Alaskan Native	4	(1.62)
Mixed Race or Other	10	(4.05)
Ethnicity		
Non-Hispanic/Latino	247	(99.2)
Hispanic/Latino	2	(0.80)
Education level		
Less than high school	78	(31.5)
High school diploma or GED	96	(38.7)
At least some college	74	(29.8)
Current marital status		
Married/living with partner	68	(28.1)
Widowed/divorced/separated/never married	174	(72.0)
Main income sources, past 6 months^a^		
Full- or part-time work	67	(26.8)
Retirement check, public assistance, or disability check	68	(27.2)
Illicit income (selling drugs, selling sex, or stealing)	54	(21.6)
Someone supports me	73	(29.2)
Housing status, past 6 months		
Unstable	142	(57.0)
Stable	107	(43.0)
Current drug of choice		
Opioids	166	(68.3)
Stimulants	60	(24.7)
Other	17	(7.00)
Receptive syringe sharing, past 30 days		
Yes	168	(73.4)
No	61	(26.2)
Injection frequency, past 30 days		
At least 2 times a day	126	(51.9)
Daily	60	(24.7)
At least weekly	28	(11.5)
At least once in the past 30 days	29	(11.9)

Data presented as N (%) unless otherwise indicated. Frequencies may not sum to total due to missing values

^a^Sums to greater than 100% as participants could select more than one response

### Frequencies and Correlations

**Fig. 1:**
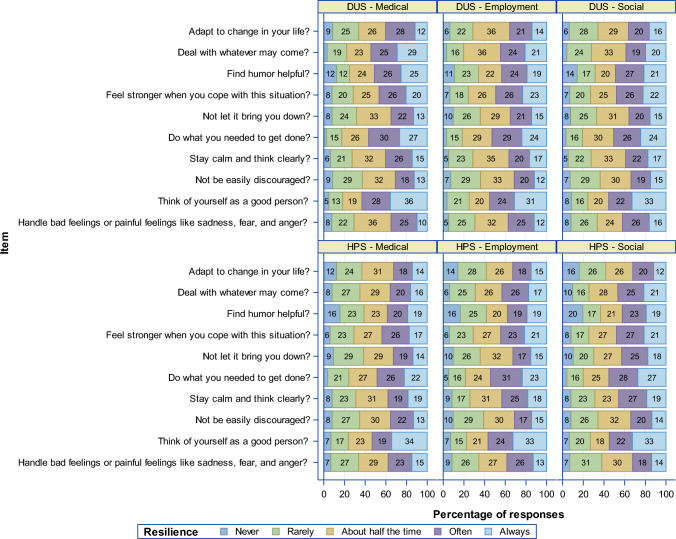
Resilience responses from n = 250 people who inject drugs (PWID) in rural Appalachian Ohio who completed a quantitative survey March–October 2023. Frequencies and percentages are presented in stacked bar charts for each item and each of the six scenarios: drug use-related stigma in medical, employment, and social contexts and HIV prevention-related stigma in medical, employment, and social contexts. Items were adapted from the 10-item Connor-Davidson Resilience Scale and followed each scenario and the phrase “After experiencing a situation like this in your daily life, how often would you be able to:”. DUS—drug use-related stigma; HPS—HIV prevention-related stigma

Resilience varied between stigmas (drug use-related and HIV prevention-related) and contexts (medical, employment, social; Fig. 1). For example, participants more frequently reported “always” being able to “deal with whatever may come” when faced with drug use-related stigma in the medical context (29%) compared to employment (21%) and social contexts (20%). For HIV prevention-related stigma, participants’ ability to “always” “deal with whatever may come” was generally lower, with corresponding frequencies of 16%, 17%, and 21%, respectively, across the contexts. Across both stigma types, “think of yourself as a good person” was the most frequently endorsed “always” response, while “find humor helpful” was the most common "never" response.

Item correlations across stigma contexts were generally moderate, indicating variation in item responses to the same item across contexts within stigma type (Table 2). Likewise, strong uniform dispersion across both mean scores (x-axis) and differences between scores (y-axis) on Bland–Altman plots suggested that responses to the same contexts are not consistently biased toward one stigma type and were capturing different types of responses (Supplemental Figures S1–S3). Thus, there was not sufficient overlap to collapse stigmas or contexts into a combined scale.

**Table 2:** Drug use-related and HIV prevention-related item correlations across stigma contexts

Stigma	Drug use-related			HIV prevention-related		
Context	Medical and employment	Medical and social	Employment and social	Medical and employment	Medical and social	Employment and social
Item	Corr^a^	Corr^a^	Corr^a^	Corr^a^	Corr^a^	Corr^a^
1. Adapt to change in your life?	0.37	0.35	0.51	0.49	0.36	0.46
2. Deal with whatever may come?	0.47	0.47	0.51	0.60	0.45	0.56
3. Find humor helpful?	0.56	0.54	0.61	0.70	0.58	0.57
4. Feel stronger when you cope with this situation?	0.54	0.46	0.49	0.41	0.47	0.43
5. Not let it bring you down?	0.32	0.42	0.46	0.36	0.39	0.47
6. Do what you needed to get done?	0.51	0.42	0.50	0.58	0.50	0.49
7. Stay calm and think clearly?	0.55	0.57	0.58	0.64	0.53	0.61
8. Not be easily discouraged?	0.47	0.40	0.46	0.55	0.48	0.49
9. Think of yourself as a good person?	0.72	0.67	0.62	0.74	0.65	0.72
10. Handle bad feelings or painful feelings like sadness, fear, and anger?	0.48	0.37	0.48	0.58	0.46	0.49

^a^Non-parametric Spearman correlations

### Scale Reliability

Cronbach’s alpha demonstrated strong internal consistency for the resilience scale across all stigmas and contexts, with all exceeding 0.80. These findings support the scale’s suitability for measuring resilience across diverse stigmas and contexts.

### Factor Analysis

EFA results on the random subsamples revealed inconsistent factor loadings between the first and second subsample across stigmas and contexts (Supplemental Tables S3–S8). Therefore, we were unable to conduct CFA with the third subsample. Inconsistency in factor structure contributed to our decision to utilize a sum of the items for each stigma source and context rather than factor scores.

With the overall sample, EFA revealed distinct item loadings on two factors across stigmas and contexts, underscoring the multidimensional nature of resilience to stigma (Tables 3, 4). For resilience to drug use-related stigma in the medical context, items like "adapt to change in your life" and “find humor helpful” loaded on Factor 1, while items like "stay calm and think clearly" and "think of yourself as a good person" loaded strongly on Factor 2. For resilience to HIV prevention-related stigma in the medical context, "deal with whatever may come" loaded on Factor 1, while "not be easily discouraged" loaded on Factor 2. These differences reinforced the importance of scenario-specific analyses to account for contextual differences in how stigma is experienced.

**Table 3:**
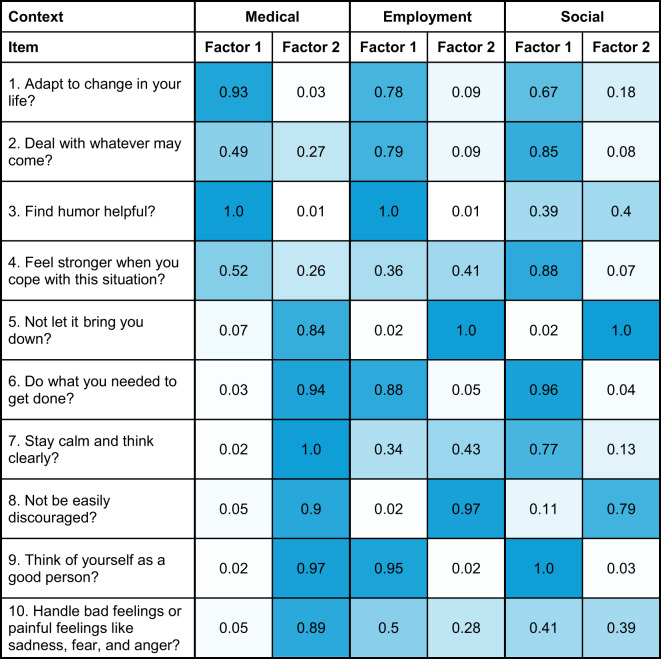
PROMAX-rotated factor loadings of scales measuring resilience to drug use-related stigma among people who inject drugs (PWID) in three contexts

**Table 4:**
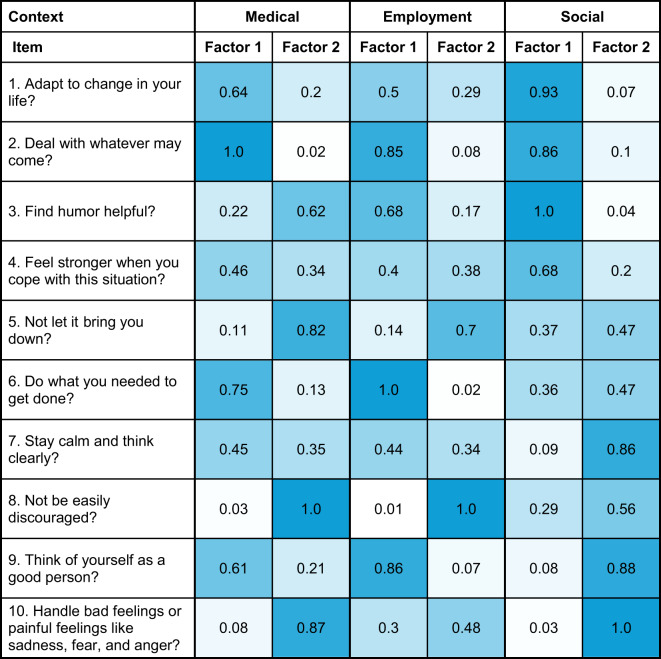
PROMAX-rotated factor loadings of scales measuring resilience to HIV prevention-related stigma among people who inject drugs (PWID) in three contexts

### PWID Resilience Scale Scores

**Fig. 2:**
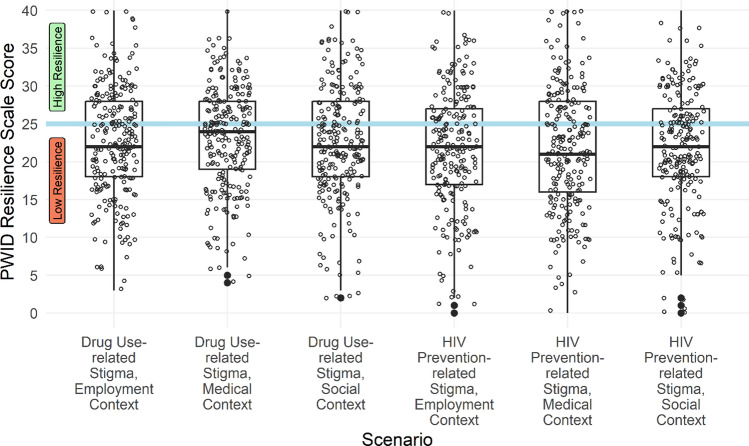
Distribution of PWID Resilience scores, by scenario with varied stigma and context, among n = 250 people who inject drugs (PWID) in rural Appalachian Ohio who completed a quantitative survey March–October 2023. Light blue line at y = 25 indicates cutoff value for “high resilience” category (≥25)

Using the 10-item sum to score each scenario, average scores ranged from 21.8 (standard deviation [SD] = 7.1) for resilience to HIV prevention-related stigma in the medical context to 23.3 (SD = 7.1) for resilience to drug use-related stigma in the medical context (Fig. 2). The next highest average scores were for resilience to drug use-related stigma in the employment context (mean = 22.7 [SD = 7.4]) and in the social context (mean = 22.7 [SD = 7.8]). Resilience to HIV prevention-related stigma averaged a score of 22.2 (SD = 8.1) in the social context and 22.1 (SD = 8.4) in the employment context.

The percentage of participants scoring high on the PWID Resilience Scale (≥25) was lowest in response to the HIV prevention-related stigma in employment context scenario (37%; n = 87) and highest in response to the drug use-related stigma in the medical context scenario (46%; n = 110 [Fig. 2]). The next highest was resilience in response to the drug use-related stigma in the employment context scenario (42%; n = 98) followed by HIV prevention-related stigma in the social context scenario (40%; n = 90), drug use-related stigma in the social context scenario (39%; n = 91), and HIV prevention-related stigma in the medical context scenario (38%; n = 87).

### Regression Analysis

In multivariable regression analyses, high resilience to stigma in specific scenarios was associated with both awareness of PrEP for HIV prevention and participation in overdose training among PWID. PWID with high resilience to HIV prevention-related stigma in medical contexts were more likely to be aware of PrEP (adjusted prevalence ratio [aPR]: 1.37; 95% CI 1.05, 1.80 [Table 5]) and to have participated in overdose training (aPR: 1.31; 95% CI 1.07, 1.61 [Table 6]). Resilience to HIV prevention-related stigma in employment and social scenarios showed nonsignificant associations for PrEP awareness (employment aPR: 1.24; 95% CI 0.93, 1.66; social aPR: 1.20; 95% CI 0.90, 1.60) and for overdose training (employment aPR: 1.08; 95% CI 0.87, 1.32; social aPR: 1.19; 95% CI 0.96, 1.46). Minimal associations were observed for drug use-related stigma scenarios. These findings suggest that high levels of resilience to stigma related to HIV prevention in medical contexts may play a critical role in promoting both PrEP awareness and overdose preparedness.

**Table 5:** Association between awareness of pre-exposure prophylaxis (PrEP) and resilience for each stigma and context

	PrEP awareness		Adjusted model^b^ PR (95% CI)
	No, DK (n = 134)	Yes (n = 109)	
High resilience^a^ to drug use-related stigma, N (%)			
Medical context	61 (49.2)	47 (43.5)	0.95 (0.72, 1.26)
Employment context	54 (43.5)	40 (38.1)	0.97 (0.72, 1.31)
Social context	46 (37.4)	41 (39.4)	1.11 (0.83, 1.49)
High resilience^a^ to HIV prevention-related stigma, N (%)			
Medical context	37 (30.8)	47 (44.3)	1.37 (1.05, 1.80)
Employment context	42 (33.9)	42 (40.4)	1.24 (0.93, 1.66)
Social context	44 (37.6)	44 (41.9)	1.20 (0.90, 1.60)

PR—prevalence ratio; CI—confidence interval; DK—do not know

^a^High resilience is scale score ≥ 25

^b^Adjusted for age category, gender of respondent, high school completion, unstable housing in past 6 months, and living with partner. Poisson distribution assumption with robust error variance

**Table 6:** Association between having received overdose training and resilience for each stigma and context

	Overdose training		Adjusted model^b^ PR (95% CI)
	No, DK (n = 95)	Yes (n = 149)	
High resilience^a^ to drug use-related stigma, N (%)			
Medical context	37 (40.7)	71 (50.0)	1.17 (0.96, 1.44)
Employment context	36 (40.4)	59 (41.5)	1.06 (0.86, 1.30)
Social context	32 (35.6)	55 (39.9)	1.12 (0.91, 1.38)
High resilience^a^ to HIV prevention-related stigma, N (%)			
Medical context	27 (30.0)	58 (42.6)	1.31 (1.07, 1.61)
Employment context	32 (36.4)	53 (37.3)	1.08 (0.87, 1.32)
Social context	32 (35.6)	57 (43.2)	1.19 (0.96, 1.46)

PR—prevalence ratio; CI—confidence interval; DK—do not know

^a^High resilience is scale score ≥ 25

^b^Adjusted for age category, gender of respondent, high school completion, unstable housing in past 6 months, and living with partner. Poisson distribution assumption with robust error variance

## Discussion

Our adaptation of the CD-RISC-10, the PWID Resilience Scale, addressed resilience to specific stigma sources in specific contexts. Our analysis of the PWID Resilience Scale provides evidence for a valid and reliable measure of resilience in marginalized populations, particularly among PWID who face multiple sources of stigma. Using the PWID Resilience Scale, we observed variability in resilience responses across contexts and stigma types, indicating resilience is context-dependent. This requires measurement tools that differentiate between distinct stigma experiences rather than relying on a one-size-fits-all approach and underscores the need for tailored interventions that address specific stigma-related barriers to healthcare engagement.

Through in-depth qualitative and cognitive interviews, we tailored a conceptually relevant resilience scale for PWID in Appalachian Ohio. Analysis of quantitative surveys demonstrated the reliability and validity of the scale. Although we did not identify a consistent factor structure within or between scenarios, the validity of our decision to instead utilize a sum of item scores is supported by Campbell-Sills and Stein’s finding of a single factor, “resilience,” of the CD-RISC-10 and is consistent with guidance for scoring the CD-RISC-10 with an item sum [35, 52]. Reliability of the PWID Resilience Scale was supported through high Cronbach’s alpha values.

Mean resilience scores in our study support the validity of the PWID Resilience Scale. PWID in our study consistently scored lower on the PWID Resilience Scale compared to resilience in the general population measured with the CD-RISC-10 [52]. We considered more than half to have “low” resilience regardless of the stigma or context described in the scenario. This is consistent with reports of lower resilience among people with substance use disorders, mental health concerns, and a history of trauma [52].

However, we observed few associations between “high” resilience and our hypothesized outcomes, increased PrEP awareness and overdose training. This suggests additional research, including longitudinal work, more diverse populations, and exploration of other resilience predictors and outcomes, is required to further assess the construct validity of the PWID Resilience Scale. Yet, patterns in our findings, including the varied results between scenarios and the association between HIV prevention-related stigma in the medical context and HIV prevention awareness and overdose preparedness, provide preliminary evidence for the construct validity of the PWID Resilience Scale.

Resilience responses to stigma experienced by PWID varied across medical, social, and employment settings, highlighting that resilience is not a uniform construct. Rather, resilience is shaped by both the type of stigma encountered and the context in which it occurs. This pattern aligns with research showing that while some PWID develop strategies to cope with drug use-related stigma in healthcare settings, HIV prevention-related stigma remains a significant and under-addressed barrier to accessing prevention services [1, 53, 54]. Low resilience to HIV prevention-related stigma is likely driven by persistent concerns about HIV disclosure, perceived judgment from healthcare providers, and the perception among many rural PWID that injection drug use is not an indicator for PrEP [1, 6, 55, 56].

Resilience to HIV prevention-related stigma in the medical context was associated with both HIV prevention awareness and overdose preparedness, suggesting that stigma-specific resilience may play a role in shaping healthcare engagement among PWID. Our findings suggest that individuals with higher resilience to stigma related to HIV prevention in medical settings are more likely to be aware of PrEP and to have participated in overdose training. This aligns with evidence that stigma resilience can facilitate access to healthcare and harm reduction services [1, 55, 57]. Future work should employ longitudinal and implementation methods to determine whether enhancing resilience in specific contexts—particularly healthcare—leads to improved outcomes. Additionally, multi-level approaches that pair resilience-building with structural interventions to reduce stigma and improve healthcare environments remain a key area for future exploration [57–60].

Our validated PWID Resilience Scale offers a preliminary tool for exploring how stigma-related resilience might inform future interventions addressing harm from stigma among PWID. Future intervention strategies might consider integrating resilience-building approaches with structural stigma reduction efforts which can enhance individual and collective agency while creating more inclusive and supportive healthcare environments for PWID. Community-driven interventions that emphasize self-efficacy, social support, and empowerment, such as peer-led support groups, harm reduction advocacy training, and leadership development programs, may foster collective resilience in the PWID community [3, 61, 62]. As existing evidence highlights, resilience alone is insufficient in addressing stigma’s structural roots; therefore, interventions must also target systemic barriers such as provider bias, discrimination, and inadequate harm reduction services [34, 59, 60]. Reducing provider-enacted stigma alongside asset-based approaches could be explored in future research to improve engagement in the HIV prevention continuum, particularly in contexts where stigma remains a key barrier [34, 63, 64]. The PWID Resilience Scale may be useful in future studies aiming to identify contexts in which increased resilience facilitates healthcare engagement and those in which systemic change may be necessary to overcome barriers.

The cross-sectional design of this study prevents determining the directionality of the relationship between resilience and HIV prevention outcomes. It remains unclear whether individuals with higher resilience were more likely to seek out information on PrEP and overdose training, or whether having knowledge and experience in these areas contributed to a greater sense of resilience in medical settings. Future longitudinal research is necessary to clarify the causal pathways between resilience, stigma, and engagement in HIV prevention. Furthermore, longitudinal studies are needed to evaluate how resilience evolves over time and whether targeted interventions enhance resilience in ways that improve engagement with HIV prevention services, including PrEP uptake and harm reduction strategies.

Additionally, since this quantitative study was conducted from a field site in a single county in rural Appalachian Ohio, which resulted in participation from residents of only one county, the findings may not fully translate to other geographic regions or PWID populations with different cultural and structural barriers. Further research should explore whether the resilience patterns observed in this study hold true in urban settings or among populations with varying levels of healthcare access and stigma exposure. Expanding research on the scale’s applicability across diverse populations and settings will be essential to assessing its generalizability, particularly in urban and non-Appalachian contexts where stigma operates differently.

Our study suggests that resilience among PWID is context-dependent and varies based on the type and setting of enacted stigma. While a single resilience scale can be adapted for multiple stigma scenarios, the differential responses across stigma types and settings indicate that collapsing resilience into a single measure may overlook important distinctions. Resilience to stigma in medical settings, particularly around PrEP, appeared to be linked to greater HIV prevention awareness and overdose preparedness, underscoring the potential benefits of interventions that both strengthen resilience among PWID and reduce stigma in healthcare settings. Additionally, our findings support the idea of tailoring resilience measures to specific stigma contexts, providing a foundation for future research to refine and expand resilience-based interventions for PWID in diverse settings.

## Supplementary Information

Below is the link to the electronic supplementary material.

Supplementary file1 (PDF 546 kb)
