# Supplementary material for: Resilience to Stigma in Medical, Social, and Employment Contexts Among People Who Inject Drugs in Rural Ohio: Adapting the 10-Item Connor-Davidson Resilience Scale
Source: AIDS Behav. 2025 Oct 28;30(3):894–906. doi: 10.1007/s10461-025-04915-4 (PMC12908228; doi:10.1007/s10461-025-04915-4)

Supplemental Material

As submitted to AIDS & Behavior for the article entitled *Resilience to Stigma in Medical, Social, and Employment Contexts Among People Who Inject Drugs in Rural Ohio: Adapting the 10-item Connor-Davidson Resilience Scale*.

Madison N. Enderle*, Rebecca H. Neiberg, Stacy M. Endres-Dighe, Nisha Gottfredson O'Shea, Vivian F. Go, William C. Miller, Kathryn E. Lancaster

*corresponding author; institutional affiliation: Wake Forest University School of Medicine, Division of Public Health Sciences, Department of Implementation Science, Winston-Salem, NC; email address: madison.enderle@advocatehealth.org

Supplemental Table 1. Excerpts of qualitative interviews conducted in rural Appalachian Ohio among people with inject drugs (PWID) from October 2021 -July 2022 which were used to develop scenarios for a scale to measure resilience among PWID. The scenarios developed covered two types of stigma encountered in three contexts and were presented to participants in each of 6 modules of the adapted PWID resilience and administered as part of a quantitative survey conducted among PWID in rural Appalachian Ohio March-October 2023.

**Drug Use-Related Stigma**		
**Context**	**Quote Describing Shared Experience**	**Scenario**
Medical	"I had horrible pain and went in [*hospital*].. as soon as I told them [*about substance use history*] it's like this switch flipped.. they went from nice and sweet to you know, instant judgement. I felt like they thought that I just wanted drugs.. They tied me and hooked me to an IV and left me in this back corner exam room. I had no call button. I couldn't get up.. couldn't get nobody's attention. They shoved me in that corner like discarded, you know, garbage."	Taylor fell and injured their shoulder while walking home. This caused them a lot of pain and they decided to go to the doctor. The doctor, staff, and nurses were efficient and treated them well until the doctor saw track marks on Taylor's arm. During the surgery, the doctor refused to give Taylor any medicine for the pain, such as a numbing shot. The nurses also spent less time with Taylor than they did before, treating them like the lowest priority, and Taylor heard them saying they thought Taylor was only in the doctor's office to get drugs.<br><br>Imagine yourself in a situation like Taylor's. After experiencing a situation like this at a doctor's, how often would you be able to:
Employment	"When my boss found out I was a heroin addict, he started treating me a little different. Wouldn't let me run the register. Always keeping his eye on me.. Wouldn't give me the hours I needed... I felt that he was biased against me. I'd been there almost 17 years and nobody treated me any different until they found out I was using... It wasn't no performance issue, somebody just told them. For 17 years, nobody knew nothing.. they labeled [*me*], once it [*the label*] was on, that's all they saw."	Alex has worked at their job for several years and has had no problems at work during that time. Recently, their boss found out that Alex injects drugs while not on the clock. Over the next few weeks, Alex noticed that they were scheduled fewer and fewer shifts and at their next performance review, they are told that they are being let go. When Alex asked why they had been fired, their boss said it had to do with the quality of their work, however, Alex felt that that their work had not been affected. They wondered if their drug use had played a role in why they had been let go.<br><br>Imagine yourself in a situation like Alex's. After experiencing a situation like this in your workplace, how often would you be able to:
Social	"I was in rehab and.. went to a church where ... everybody knows everybody. I go out there and .. they know I'm from the rehab... all these people were looking at me and I felt like my track marks had like neon signs around them. I felt very uncomfortable."	Jordan has been injecting drugs for the past few years. People in their community know about Jordan's drug use and Jordan can often feel others staring at them or talking about them behind their back when they're out getting groceries, going to church, or are outside the house.<br><br>Imagine yourself in a situation like Jordan's. After experiencing a situation like this in your daily life, how often would you be able to:

HIV Prevention-Related Stigma

**Context**	**Quote Describing Shared Experience**	**Scenario**
Medical	“>>I just talked to my doctor [*about PrEP*], and you know, she’s sort of… like you can do that but it’s like I have to do it [*on their own without physician’s help*]. “<br><br>“>>So, you've wanted to take it [*PrEP*], but you haven't been able to get it, even when you tell the physician..?”<br><br>“>> Yes.”	Kennedy has injected drugs for the past few years and recently decided to start PrEP. Kennedy told their doctor that they had learned more about PrEP and wanted to start it, but the doctor refused. They later overheard the doctor tell the nurse that Kennedy wasn't capable of remembering to take a pill each day and that PrEP would just encourage them to engage freely in risky activities.<br><br>Imagine yourself in a situation like Kennedy. After experiencing a situation like this, how often would you be able to:
Employment	“>> A lot of people are going to be hard pressed to go in and get a treatment like that and let everybody know about it. Because then you're going to be called a fag, or you're going to be shunned, you know, and people aren't going to want nothing to do with you, they're not going to want you to be around them.”	Blake has worked successfully as a line cook for years. When not on the clock, they inject drugs. Blake normally takes PrEP at night, but a long shift led to them taking the anti-HIV medication at work. A co-worker noticed "HIV" on the prescription label, assumed Blake was HIV positive, and refused to work with them. The manager was informed. Now Blake is no longer scheduled as a cook and receives few shifts.<br><br>Imagine yourself in a situation like Blake. After experiencing a situation like this, how often would you be able to:
Social	“>> Are there some reasons that you might not want to use PrEP?”<br><br>“>> Embarrassment of people knowing. Like, why is she taking that, you know? People know what I do, but, you know, just people. People judge, are quick to judge, real quick to judge.”	Sam has been injecting drugs for several years. If Sam doesn't have clean syringes, they'll occasionally use after others. Sam recently started taking PrEP as precautionary measure to protect themselves from HIV. A friend noticed Sam taking the pill and started spreading rumors that Sam's using PrEP because Sam and their partner are sleeping around.<br><br>Imagine yourself in a situation like Sam's. After experiencing a situation like this in your daily life, how often would you be able to:

Supplemental Table 2. Connor-Davidson Resilience Scale (CD-RISC) domains of the CD-RISC-10 items and adapted People Who Inject Drugs (PWID) Resilience Scale items.

Original CD-RISC Domain	CD-RISC-10 Item	PWID Resilience Scale Item
Notion of personal competence, high standards, and tenacity (Grit)	Can achieve goals despite obstacles	Do what you needed to get done?
	Not easily discouraged by failure	Not be easily discouraged?
	Thinks of self as strong person	Think of yourself as a good person?
Trust in one's instincts, tolerance of negative affect and strengthening effects of stress (Unflappability)	Tries to see humorous side of problems	Find humor helpful?
	Coping with stress can strengthen me	Feel stronger when you cope with this situation?
	Can stay focused under pressure	Stay calm and think clearly?
	Can handle unpleasant feelings	Handle bad feelings or painful feelings like sadness, fear, and anger?
Positive acceptance of change and secure relationships (Acceptance)	Able to adapt to change	Adapt to change in your life?
	Can deal with whatever comes	Deal with whatever may come?
	Tends to bounce back after illness or hardship	Not let it bring you down?

Supplemental Table 3. PROMAX-rotated factor loadings of scales measuring resilience to drug use-related stigma among people who inject drugs (PWID) in a medical context among two random subsamples.

Item	Sample 1 (n=76)		Sample 2 (n=80)	
	Factor 1	Factor 2	Factor 1	Factor 2
1. Adapt to change in your life?	0.35	0.65	0.26	0.53
2. Deal with whatever may come?	0.56	0.49	0.49	0.27
3. Find humor helpful?	0.06	0.63	0.15	0.57
4. Feel stronger when you cope with this situation?	0.24	0.67	0.12	0.81
5. Not let it bring you down?	0.53	0.13	0.50	0.38
6. Do what you needed to get done?	0.55	0.31	0.64	0.15
7. Stay calm and think clearly?	0.86	0.08	0.53	0.36
8. Not be easily discouraged?	0.66	0.23	0.46	0.31
9. Think of yourself as a good person?	0.58	0.21	0.62	-0.01
10. Handle bad feelings or painful feelings like sadness, fear, and anger?	0.60	0.24	0.62	0.31

Supplemental Table 4. PROMAX-rotated factor loadings of scales measuring resilience to drug use-related stigma among people who inject drugs (PWID) in an employment context among two random subsamples.

Item	Sample 1 (N = 78)		Sample 2 (N = 80)	
	**Factor 1**	**Factor 2**	**Factor 1**	**Factor 2**
1. Adapt to change in your life?	0.59	0.11	0.59	0.20
2. Deal with whatever may come?	0.64	0.43	0.45	0.53
3. Find humor helpful?	0.47	0.22	0.35	0.15
4. Feel stronger when you cope with this situation?	0.65	0.33	0.63	0.10
5. Not let it bring you down?	0.15	0.69	0.61	0.09
6. Do what you needed to get done?	0.60	0.37	0.14	0.72
7. Stay calm and think clearly?	0.47	0.59	0.61	0.34
8. Not be easily discouraged?	0.26	0.89	0.52	0.19
9. Think of yourself as a good person?	0.68	0.11	0.13	0.72
10. Handle bad feelings or painful feelings like sadness, fear, and anger?	0.63	0.55	0.44	0.43

Supplemental Table 5. PROMAX-rotated factor loadings of scales measuring resilience to drug use-related stigma among people who inject drugs (PWID) in a social context among two random subsamples.

Item	Sample 1 (N = 76)		Sample 2 (N = 81)	
	Factor 1	Factor 2	Factor 1	Factor 2
1. Adapt to change in your life?	0.48	0.39	0.75	0.39
2. Deal with whatever may come?	0.69	0.55	0.64	0.09
3. Find humor helpful?	0.21	0.69	0.31	0.21
4. Feel stronger when you cope with this situation?	0.45	0.56	0.56	0.06
5. Not let it bring you down?	0.39	0.49	-0.01	0.96
6. Do what you needed to get done?	0.78	0.26	0.54	0.16
7. Stay calm and think clearly?	0.74	0.41	0.56	0.26
8. Not be easily discouraged?	0.36	0.65	0.17	0.40
9. Think of yourself as a good person?	0.58	0.26	0.39	0.08
10. Handle bad feelings or painful feelings like sadness, fear, and anger?	0.64	0.39	0.30	0.48

Supplemental Table 6. PROMAX-rotated factor loadings of scales measuring resilience to drug use-related stigma among people who inject drugs (PWID) in a medical context among two random subsamples.

Item	Sample 1 (N = 76)		Sample 2 (N = 83)	
	Factor 1	Factor 2	Factor 1	Factor 2
1. Adapt to change in your life?	0.29	0.70	0.40	0.43
2. Deal with whatever may come?	0.52	0.56	0.83	0.11
3. Find humor helpful?	0.28	0.67	0.07	0.52
4. Feel stronger when you cope with this situation?	0.49	0.68	0.32	0.42
5. Not let it bring you down?	0.65	0.39	0.25	0.65
6. Do what you needed to get done?	0.56	0.54	0.81	0.19
7. Stay calm and think clearly?	0.65	0.58	0.52	0.37
8. Not be easily discouraged?	0.58	0.33	0.21	0.61
9. Think of yourself as a good person?	0.38	0.61	0.45	0.25
10. Handle bad feelings or painful feelings like sadness, fear, and anger?	0.89	0.31	0.21	0.65

Supplemental Table 7. PROMAX-rotated factor loadings of scales measuring resilience to drug use-related stigma among people who inject drugs (PWID) in an employment context among two random subsamples.

Item	Sample 1 (N = 76)		Sample 2 (N = 80)	
	Factor 1	Factor 2	Factor 1	Factor 2
1. Adapt to change in your life?	0.28	0.59	0.50	0.48
2. Deal with whatever may come?	0.61	0.50	0.68	0.14
3. Find humor helpful?	0.12	0.77	0.42	0.10
4. Feel stronger when you cope with this situation?	0.58	0.49	0.69	0.18
5. Not let it bring you down?	0.57	0.34	0.16	0.75
6. Do what you needed to get done?	0.57	0.42	0.62	0.40
7. Stay calm and think clearly?	0.85	0.04	0.62	0.39
8. Not be easily discouraged?	0.65	0.31	0.17	0.65
9. Think of yourself as a good person?	0.67	0.35	0.42	0.28
10. Handle bad feelings or painful feelings like sadness, fear, and anger?	0.76	0.26	0.30	0.53

Supplemental Table 8. PROMAX-rotated factor loadings of scales measuring resilience to drug use-related stigma among people who inject drugs (PWID) in a social context among two random subsamples.

Item	Sample 1 (N = 75)		Sample 2 (N = 78)	
	Factor 1	Factor 2	Factor 1	Factor 2
1. Adapt to change in your life?	0.74	0.08	0.65	0.32
2. Deal with whatever may come?	0.55	0.48	0.62	0.34
3. Find humor helpful?	0.68	0.23	0.57	-0.02
4. Feel stronger when you cope with this situation?	0.56	0.43	0.61	0.27
5. Not let it bring you down?	0.43	0.50	0.54	0.27
6. Do what you needed to get done?	0.28	0.64	0.59	0.29
7. Stay calm and think clearly?	0.33	0.79	0.54	0.56
8. Not be easily discouraged?	0.34	0.60	0.59	0.27
9. Think of yourself as a good person?	0.56	0.44	0.13	0.69
10. Handle bad feelings or painful feelings like sadness, fear, and anger?	0.07	0.82	0.25	0.66

Supplemental Figure 1. Bland-Altman Plots between medical, employment, and social contexts of drug use-related stigma scenarios. **A)** Difference of medical context resilience score less employment context resilience score plotted against the mean resilience score of medical and employment contexts. **B)** Difference of medical context resilience score less social context resilience score plotted against the mean resilience score of medical and social contexts. **C)** Difference of social context resilience score less employment context resilience score plotted against the mean resilience score of social and employment contexts.

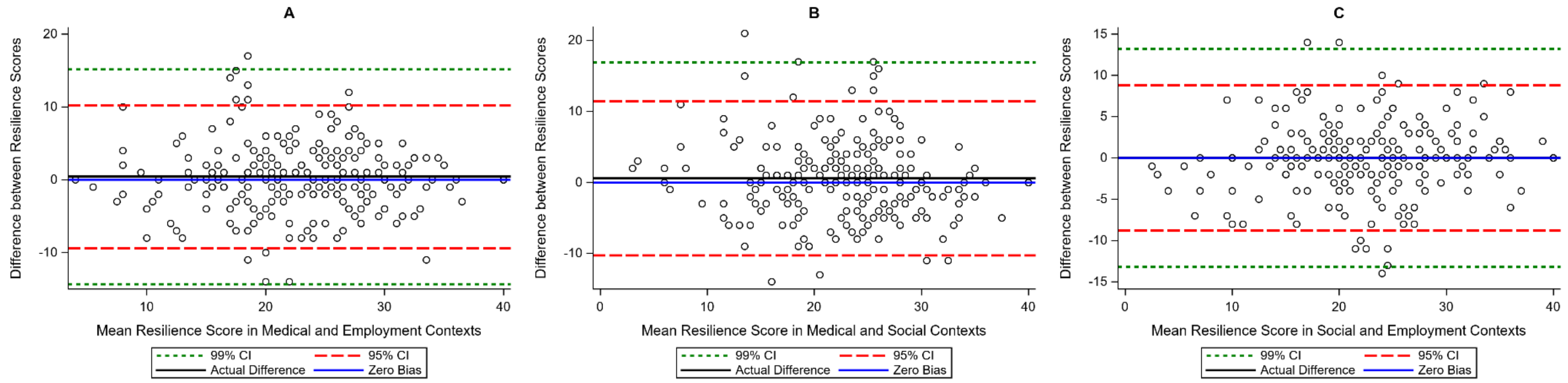

Supplemental Figure 2. Bland-Altman Plots between medical, employment, and social contexts of HIV prevention-related stigma scenarios. **A)** Difference of medical context resilience score less employment context resilience score plotted against the mean resilience score of medical and employment contexts. **B)** Difference of social context resilience score less medical context resilience score plotted against the mean resilience score of social and medical contexts. **C)** Difference of social context resilience score less employment context resilience score plotted against the mean resilience score of social and employment contexts.

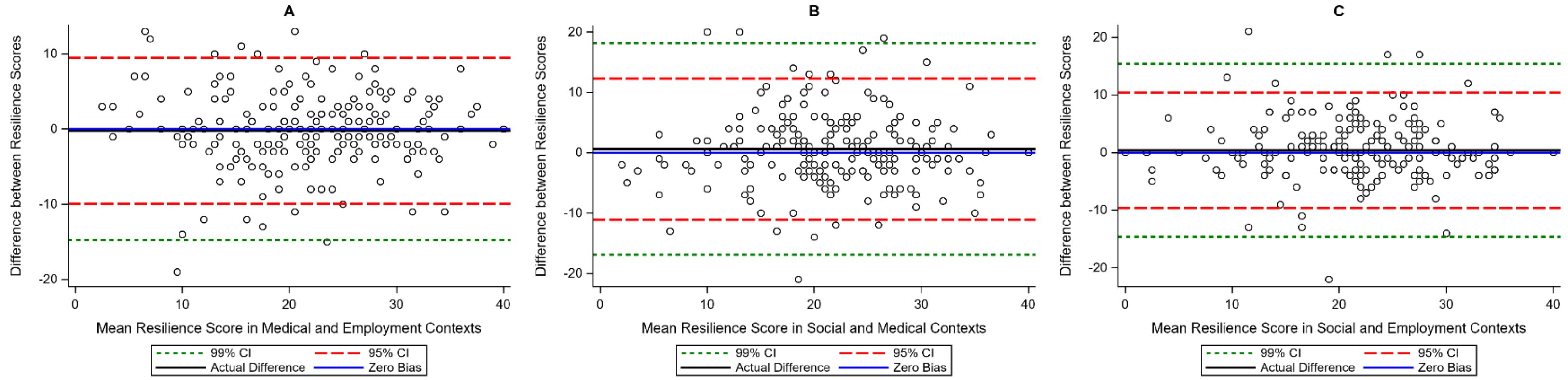

Supplemental Figure 3. Bland-Altman Plots between resilience to drug use-related stigma (DUS) and HIV prevention-related stigma (HPS) within different contexts. Difference of DUS resilience score less HPS resilience score plotted against mean resilience score. **A)** Social context. **B)** Employment context. **C)** Social context.

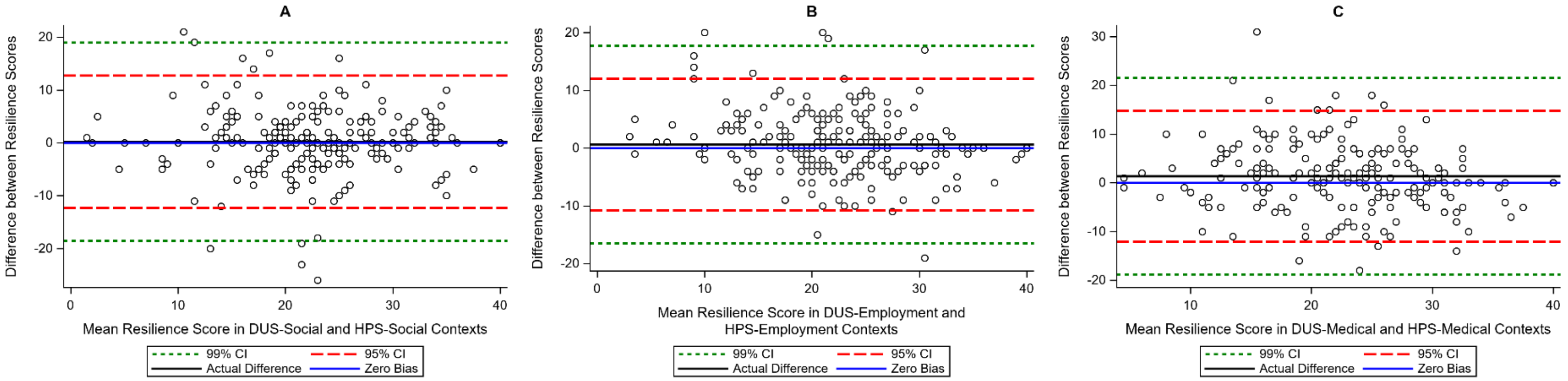

Supplement: Supplementary file 1 — Supplementary file1 (PDF 546 kb) [file 10461_2025_4915_MOESM1_ESM.pdf]
